# Targeting Executive Dysfunction in Post-Stroke Rehabilitation Via Synergism of Cognitive-Behavioral Therapy and Methylphenidate

**DOI:** 10.1192/j.eurpsy.2026.11609

**Published:** 2026-07-31

**Authors:** O. Scheuermann, B. Carr

**Affiliations:** 1 College of Medicine; 2 Department of Psychiatry, University of Florida, Gainesville, United States

## Abstract

**Introduction:**

Executive dysfunction, including impaired attention, working memory, and cognitive flexibility, is common after stroke and directly impairs recovery, independence, and quality of life. While cognitive-behavioral therapy (CBT) improves metacognitive strategies, it requires sustained attentional engagement, which is often blunted post-stroke. Methylphenidate, a dopaminergic-noradrenergic agent, enhances prefrontal cortex function and may potentiate CBT efficacy by improving attentional and motivational drive.

**Objectives:**

To evaluate the mechanistic rationale and preliminary evidence supporting the combined use of CBT and methylphenidate in post-stroke patients with executive dysfunction.

**Methods:**

A literature review was performed on clinical studies and neuroimaging investigations in PubMed and Embase involving post-stroke CBT, methylphenidate use, or both. We focused on cognitive domains affecting treatment timing, neural correlates of response, and functional outcome.

**Results:**

CBT improves executive function post-stroke via structured goal-setting, cognitive reframing, and self-monitoring, primarily engaging the dorsolateral prefrontal cortex. Methylphenidate increases synaptic dopamine and norepinephrine, enhancing signal-to-noise ratios in prefrontal and anterior cingulate regions; networks critical for attention and behavioral regulation. Pilot studies show that methylphenidate improves working memory and processing speed, while also increasing engagement in cognitive training. Though combined protocols are scarce, emerging evidence suggests that pharmacologic enhancement may optimize cognitive receptivity during therapy, particularly in patients with attentional fatigue or apathy.

**Conclusions:**

CBT and methylphenidate target complementary aspects of post-stroke executive dysfunction: top-down strategy implementation and bottom-up neurochemical activation, respectively. Integrated treatment protocols merit clinical investigation as a means of amplifying neurocognitive recovery in stroke survivors with frontal-executive deficits.

**Disclosure of Interest:**

None Declared

